# Neural evidence for attentional resource allocation to postural control using brain-body imaging

**DOI:** 10.1016/j.bbr.2025.115716

**Published:** 2025-07-05

**Authors:** Sodiq Fakorede, Fatimah Alkhameys, Ke Liao, Laura Martin, Hannes Devos

**Affiliations:** a Department of Physical Therapy, Rehabilitation Science, and Athletic Training, University of Kansas Medical Center, Kansas, KS, USA; b Hoglund Biomedical Imaging Center, University of Kansas Medical Center, Kansas, KS, USA; c Department of Population Health, University of Kansas Medical Center, Kansas, KS, USA

**Keywords:** Postural control, Cognitive workload, EGG, ERP, Multivariate analysis, Auditory oddball

## Abstract

**Objective:**

To examine whether bipedal stance (quiet standing) requires more attentional resources than sitting during a concurrent cognitive task.

**Methods:**

126 adults completed an auditory oddball task in both standing and sitting positions. Neural activity was recorded using electroencephalography (EEG) in a mobile brain-body imaging setup. Univariate analysis compared P3 event-related potentials (ERP) across conditions during frequent, rare, and novelty stimuli. Multivariate pattern analysis (MVPA) with a support vector machine (SVM) was used to decode ERP signals between standing and sitting conditions.

**Results:**

The P3b component, associated with conscious stimulus evaluation, showed lower amplitude in the standing condition at the parietal channel (Pz). No significant differences were found in the P3a component at the frontal channel (Fz). MVPA effectively distinguished ERP signals between standing and sitting conditions from 100 ms to 800 ms post-stimulus.

**Conclusions:**

Upright stance requires more attentional resources, diverting focus from concurrent cognitive tasks that require conscious decision making. Machine learning models reveal that quiet standing also influences sensory and motor-related neural activity, indicating that postural demands not only impact neural activity related to cognitive performance, but also motor and sensory processing.

## 1. Introduction

### 1.1. Cortical involvement in postural control

The transition from a quadrupedal to a bipedal stance marked a pivotal milestone in human evolution [1]. The ability to balance on two feet fundamentally altered mobility, freed the hands for tool use, and enabled the development of complex social behaviors [2]. Postural control is essential for maintaining balance while standing upright and involves keeping the body’s center of mass over its base of support or, more generally, within the limits of stability [3]. Initially, postural control was considered an automated activity [4], governed by reflexive and muscular mechanisms to anticipate and correct changes in the body’s center of mass [5]. By the end of the 20th century, postural control was recognized as an intricate interplay of spinal and supraspinal functions to manage the constant and dynamic interaction of sensory inputs and motor outputs involved in maintaining balance [6]. Functional magnetic resonance imaging (fMRI) studies revealed several subcortical brain areas, including the brain stem, cerebellum, basal ganglia, and thalamus, to plan, anticipate, and execute internal models for the execution of postural control [7]. These fMRI studies also provided increasing evidence of cortical involvement in postural control [7]. This cortical engagement is crucial for the allocation of the necessary attentional resources to changes in postural demand [8]. Even quiet standing requires attention to anticipate, react, or adapt to the visual, proprioceptive, and vestibular inputs [9]. Therefore, the benefits of upright stance in human evolution may have come with the cost of requiring additional attentional resources for maintaining postural balance.

### 1.2. Electro-encephalography mobile brain-body imaging for postural control

Mobile brain-body imaging (MoBI) vastly improved the understanding of the cortical involvement in the control and adjustment of posture. Unlike fMRI, MoBI imaging enables real-time, continuous recording of cortical activity in response to changes in postural control. Functional near-infrared spectroscopy (fNIRS) is a MoBI tool that measures changes in oxygenated and deoxygenated hemoglobin concentrations in specific regions of interest [10]. fNIRS studies have demonstrated that the interplay between sensory and motor systems to regulate postural control requires intact attentional resources [11]. The attentional resources needed for postural control have been investigated during dual-task paradigms where participants are asked to stand while performing an additional cognitive task. During dual-tasking, participants exhibit increased activation in the prefrontal cortex, highlighting a need for higher-order cognitive processes such as executive function, working memory, and spatial awareness in the regulation of postural control [12].

While fNIRS provides the spatial resolution needed to locate the cortical areas involved in postural control through hemodynamic changes, electro-encephalography (EEG) MoBI directly captures the neural processes of postural control with great temporal resolution. Event-related potentials (ERP) offer the advantage of directly linking specific cortical responses to discrete stimuli. Some ERP studies have focused on the first negative peak (N1) and the second positive peak (P2) of the ERP wave in response to balance perturbations [13]. These peaks provide insight into the somatosensory processes involved in maintaining balance. The P3 component, particularly the P3b subcomponent, is associated with the allocation of attentional resources and the evaluation and categorization of task-relevant stimuli [14]. The P3 reflects higher-order cognitive processes such as context updating and decision-making, which are crucial for integrating and responding to complex stimuli [15]. A systematic review of 29 studies evaluated the impact of performing a balance and cognitive task at the same time (dual-task) on the P3 ERP [16]. Most studies showed a lower P3 amplitude in walking compared to sitting conditions, demonstrating a higher need for cognitive resources for walking compared to sitting [16]. However, there is a surprising paucity of evidence on how a seemingly effortless act of standing affects neural activity compared to sitting [17]. Quiet stance with the eyes open on a firm surface allows for the integration of visual, proprioceptive, and vestibular systems to generate the motor outputs for balance. This integration requires additional resources, potentially taking away attentional focus from the cognitive dual-task. Evaluating the P3 under both single cognitive tasks and dual cognitive-posture tasks will not only identify the resources required to maintain balance, dissecting the P3 component into the conscious P3b and the automated P3a will reveal the specific cognitive processes immediately impacted by postural control.

### 1.3. Advantages of Multivariate Pattern Analysis

Univariate analyses of ERP amplitude and latency average cortical activity over a designated time window across many trials, on a single channel or cluster of channels. Multivariate pattern analysis (MVPA) of ERP signals assess how the information content of the neural signals differs across classes [18], by dissociating the magnitude of the neural response from the decodable information content [19]. Unlike univariate ERP, MVPA of ERP uses machine learning algorithms to predict the classes based on the neural activity recordings of all channels at each time point for each participant separately. MVPA offers a more holistic view of neural dynamics by leveraging the spatial distributions of the ERP signals that contribute to cognitive and postural control processes. Therefore, it is possible that the MVPA discerns complex patterns in the ERP between sitting and standing that may not be captured by univariate analyses [20]. MVPA approaches have been used to accurately distinguish ERP patterns for face recognition [21], emotion regulation [22], performance monitoring[23], and conflict resolution [24], but have not been applied in postural balance tasks.

### 1.4. Aim of the current study

The aim of the current study was to evaluate the effect of postural balance on neural activity during a dual-task paradigm. The effects of dual-tasking on balance outcomes are usually investigated by manipulating the sensory inputs (e.g., standing with eyes closed, standing on foam) or by modifying the domain or difficulty of the cognitive task [25]. Very few dual-task studies have compared the effects of changing body position from sitting to standing on cognitive behavioral outcomes [26–28]. Some studies indicated better cognitive performance in standing and others in sitting, depending on the cognitive domain tested [26–28]. In an auditory working memory task, Lajoie and colleagues demonstrated that cognitive performance in sitting was better than in standing [29]. We therefore hypothesized that a simple standing task will decrease the P3 amplitude of an auditory working memory task compared to sitting. Second, we hypothesized that MVPA will successfully decode the ERP activity during a cognitive task while quiet standing from a cognitive task while seated.

## 2. Methods

### 2.1. Participants

Participants were recruited from 03/07/2023–07/30/2024 through social media advertisements and word-of-mouth. Of the 238 potential participants who were contacted, 126 who met the eligibility criteria were enrolled. Inclusion criteria were: (1) age between 18 and 90 years; (2) Montreal Cognitive Assessment (MoCA) scores of 26 and above; (3) ability to stand for 2 min; and (4) follow simple instructions in English. Exclusion criteria were: (1) neurological disorders or musculoskeletal disorders that affect balance; (2) subjective cognitive decline or diagnosed cognitive impairment; and (3) uncorrected hearing loss. This cross-sectional study was approved by the University of Kansas Medical Center Institutional Review Board (#00149615, #00150307, and #00150444). All participants signed the informed consent form.

### 2.2. Protocol

#### 2.2.1. Collection of demographic information

Demographic information such as age, sex, race, and education were recorded through a questionnaire designed in REDCap (Table 1). The MoCA was administered to screen for cognitive function on a scale ranging from 0 to 30 [30].

#### 2.2.2. Auditory oddball task

Participants completed a cognitive task in both standing and sitting positions, with the order of presentation randomized [14]. This auditory novelty oddball task consisted of a series of 50 sounds, played through a Bluetooth speaker that was located on the floor right in front of the participant. Of those stimuli, 40 (80 %) included frequent, low pitched sounds (600 Hz pure tone burst; 500 ms duration [31]; rise/fall time of 10 ms), 5 (10 %) rare, high pitched sounds (1200 Hz pure tone burst; 500 ms duration [31]; rise/fall time of 10 ms) and 5 (10 %) novelty sounds (159 – 399 ms duration) that included inanimate sounds (music; video game), animate sounds (cat; bird), or human sounds. The inter-stimulus interval (ISI) was 1900 ms with a jitter of ± 200 ms. Although the trials were presented at random, trials were organized so that every rare or novelty stimulus was presented after a frequent stimulus. The total duration of the task was about 120 s. The stimuli were created in E-Prime 3.0. Participants used the bottom button of a Bluetooth clicker to respond to frequent stimuli, the top button to respond to rare stimuli, and were asked to ignore the novelty stimuli [32]. Before the formal trials, each participant completed a brief practice block to verify audibility and correct tone discrimination; all participants met this criterion. All participants were instructed to respond using their dominant hand to minimize variability in motor execution; only two participants were left-handed. Although requesting a button press to respond to the frequent and rare trials may introduce motor confounds to the P3 ERP [33], the ability to record behavioral measures (such as reaction times and false alarms), the real-time assessment of engagement to the cognitive task by the examiner, and the increased task saliency are three advantages that outweigh the potential introduction of motor-related potentials to the P3. Behavioral outcomes included response time and number of correct responses. Instructions were given to pay equal attention to the balance and cognitive task.

#### 2.2.3. Electroencephalography recording and processing

Participants were fitted a MoBI EXG net (Mentalab, Munich, Germany) that records at 250 Hz. Eight dry small brush electrodes were placed according to the 10–20 classification at channel locations Fz, FCz, Cz, CPz, Pz, Oz, Fpz, and Fp1. A wet, flat reference electrode was used to capture activity at the mastoid. Impedance was kept below 30 kΩ. The EEG data, captured with the Mentalab Explore Pro, and the behavioural data (response time and accuracy), captured with Eprime, were synchronized with the Lab Streaming Layer [34]. EEG data were processed in MATLAB R2023b [35], EEGLab v2024 [36], and ERPLab v11.03 [37]. Continuous EEG were first filtered between 0.1 and 30 Hz in EEGLab. We then applied artificial space reconstruction (ASR) to the filtered data to remove artifacts using the clean_raw data function. Event-related potentials were extracted using ERPLab. Epochs were extracted with a prestimulus baseline correction and a time range between −200 ms and 800 ms. Artifacts in the ERP epochs with a threshold ±100 μV in any channel were removed. Each participant had 50 epochs for both sitting and standing conditions, yielding 6300 epochs per condition across the study. An average of 14.59 % of epochs (919 out of 6300) were rejected in the standing condition, compared to 3.41 % (215 out of 6300) in the sitting condition.

#### 2.2.4. Standing and sitting conditions

**Fig. 1.**
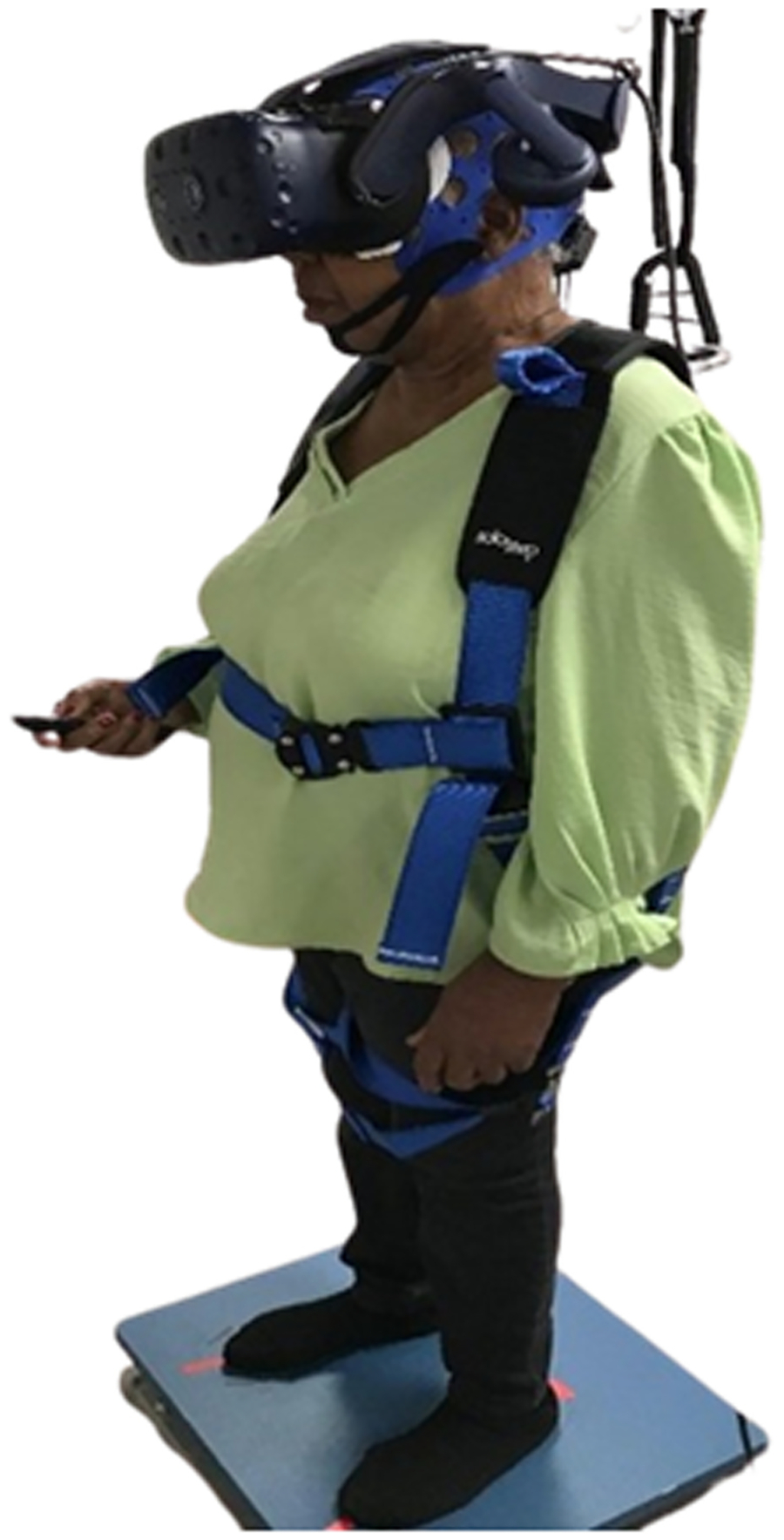
Experimental setup of the standing condition. Participants completed quiet standing (condition 1) of the Virtual Reality Sensory Organization Test (VR-SOT) using the Virtual Reality Comprehensive Balance Assessment and Training (VR-COMBAT) system (Participant is bending the right elbow for demonstration purposes to show the clicker). Participant signed photo and video release statement.

For the standing condition, participants completed the quiet standing (condition 1) of the Virtual Reality Sensory Organization Test (VR-SOT) in the Virtual Reality Comprehensive Balance Assessment and Training (VR-COMBAT) system (Fig. 1) [38]. This condition involves participants standing on a firm platform (force plate, AMTI), shoes removed and feet apart, while wearing a VR headset (HTC Vive Pro Eye). The VR environment displays a three-dimensional room with triangles depicted on the walls to enable participants to focus their eyes. Participants were instructed to keep their eyes open during the auditory oddball task, hold the clicker in their right hand, and keep both arms relaxed aside the trunk. A safety harness was used to prevent participants from falling. A spotter also ensured safety of the participants.

The sitting condition involved participants sitting in an armchair. Participants were instructed to look straight ahead for two minutes while responding to the oddball task and pressing the buttons with their right hand.

### 2.3. Analyses

#### 2.3.1. Univariate analysis

We extracted the stimulus-evoked P3 of the frequent responses, rare responses, and the difference between rare – frequent responses. We calculated the P3b at channel Pz as the P3b is largest at the parietal channels during oddball tasks [15]. Since the P3a has a more frontal distribution, we extracted P3a at the frontal channel Fz from the novelty responses [15]. We measured the mean amplitude and the 50 % fractional area latency in these time intervals. We compared the P3 amplitudes between the sitting and standing conditions using signed rank tests, given the non-normal distribution, and the P3 latencies between the sitting and standing conditions using dependent t-tests. We adjusted for biological variables such as age and sex in linear mixed models with subject included as a random effect to account for intra-individual variability. We also compared the relationship between behavioral reaction time and P3 ERP using similar linear mixed models. The interaction effect of behavioral reaction time by condition was calculated to estimate differences in association between behavioral reaction time and P3 ERP in sitting and standing conditions. We compared behavioral responses such as accuracy using signed rank tests and reaction times using dependent t-tests. Two-tailed p values of < 0.05 were considered significant. These analyses were calculated using SAS 9.4.

#### 2.3.2. ERP decoding analysis

The Multivariate Pattern Classification toolbox in ERPLab was used to decode the ERP. Decoding was performed separately for each participant at each time sample. We performed two multivariate pattern analyses (MVPA) using the support vector machine (SVM) algorithm. The two classes that were decoded in the first MVPA were the frequent trials of the sitting condition and the frequent trials of the standing condition. We could not decode the MVPA of the rare and novelty ERP separately, since these trials only had five trials per condition. However, a second MVPA merged all trials (frequent, rare, and novelty) of the sitting condition and all trials (frequent, rare, and novelty) of the standing condition into two classes for decoding. We used the MATLAB function fitcsvm() to train the SVM, and the MATLAB function predict() to test the decoder. As in previous studies [39,40], we used a 3-fold cross-validation to validate the effectiveness of the classification. We equalized trials across classes (conditions) and across files. Only trials with 90 % or more clean trials were included in the MVPA. Thus, atasets with fewer than 36 epochs of frequent stimuli were excluded, leaving 12 random trials per averaged ERP for each fold. Similarly, datasets with fewer than 45 combined epochs of frequent, rare, and novelty stimuli were removed, leaving 15 random trials per averaged ERP for each fold with the merged trials. Out of the 126 datasets, we entered 89 (70.63 %) in the SVM analysis of the frequent trials and 88 (69.84 %) in the SVM analysis of the merged trials. The SVM analysis was reiterated 100 times. For each iteration, we re-randomized the assignment of trials to averages and trained new SVM. Decoding accuracy was defined as the proportion of correctly classified test cases. This procedure was applied separately to each time point in the ERP waveform, with each participant being decoded independently, resulting in a separate decoding accuracy value at each time point for each participant. Finally, the grand average of the individual decoded sets was calculated to produce an average decoding accuracy for each time point in the waveform. With two classes in the SVM and an equal number of training cases for both classes, the chance decoding accuracy was 0.5 at each time point.

#### 2.3.3. Statistical analysis of decoding accuracy

A non-parametric, cluster-based permutation approach was employed to compare decoding accuracy against chance at each time point using MATLAB [40–42]. This method accommodates non-normal distribution of decoding accuracy corrects for multiple comparisons, and accounts for noise autocorrelation in EEG data [43].

To determine whether decoding accuracy at each time point post-stimulus onset exceeded chance level (50 %), one-tailed t-tests were conducted. Since the SVM does not yield meaningful below-chance decoding, a one-tailed *t*-test was deemed appropriate. Significant consecutive time points (p < 0.05) were identified to form clusters, and the t-scores within each cluster were summed to calculate a cluster-level t-mass. This t-mass was then compared against a null distribution generated through permutation analysis.

For the permutation analysis, the true labels (standing and sitting) in the test data were randomly permuted before computing decoding accuracy, rather than permuting data labels and then performing the training-testing procedure [40]. This expedited the construction of the null distribution while ensuring that the decoding accuracy remained at chance level, accounting for temporal autocorrelation in the data. After computing decoding accuracy with permuted labels, a 5-point running average filter was applied, consistent with the actual decoding accuracy. Consecutive time points were then clustered as described earlier. The cluster mass was set to zero for permutation trials that did not identify significant clusters. In cases where multiple significant clusters were found, the mass of the largest cluster was used.

This permutation process was repeated 1000 times with randomly permuted true labels, constructing an empirical null distribution of the cluster-level t-mass with a resolution of p = 0.001. P values for each cluster in the actual dataset were determined by comparing the observed t-masses to the percentiles of the null distribution. A p value of < 0.001 was reported if the observed cluster-level t-mass exceeded the maximum t-mass from the permuted clusters. The null hypothesis was rejected, and decoding was deemed above chance when a cluster-level t-mass fell within the top 95 % of the null distribution (alpha = 0.05). Finally, a confusion matrix was generated by collapsing predicted stimulus labels across all time points within the P3 window (250–550 ms) and across the significant cluster window identified by MVPA.

#### 2.3.4. ERP Data Quality Analysis

To evaluate data quality in our study, we calculated the analytic standardized measurement error (aSME) for the P3 mean amplitude at channels Pz and Fz [44]. The aSME was determined by first computing the mean amplitude for each individual trial within the 250–550 ms time window, then calculating the standard deviation of these values, and finally dividing by the square root of the total number of trials [44].

The aSME method is not appropriate for the 50 % fractional area latency, because the average of single-trial latencies does not match the latency derived from the averaged ERP waveform. Therefore, we applied a bootstrapping approach to estimate the SME for this measure. In this bootstrapped SME (bSME), trials from the merged conditions (including frequent, rare, and novelty stimuli) were randomly resampled with replacement, and new ERP waveforms were created for each iteration. This process was repeated 10,000 times for both the standing and sitting conditions. The bSME was then obtained by calculating the standard deviation of these 10,000 latency values [44]. We compared average SME values between standing and sitting conditions using dependent t-tests in SAS 9.4.

## 3. Results

### 3.1. Behavioral performance

Table 2 summarizes the behavioral performance in response to frequent and rare oddball stimuli under standing versus sitting conditions. There were no significant differences in accuracy for frequent stimuli (p = 0.07) or in reaction time for frequent stimuli (p = 0.11). Accuracy for rare stimuli also did not differ between conditions (p = 0.55). The reaction time to rare stimuli was longer in the standing versus sitting condition, but did not reach significance (p = 0.06).

### 3.2. Results of the univariate analyses

**Fig. 2.**
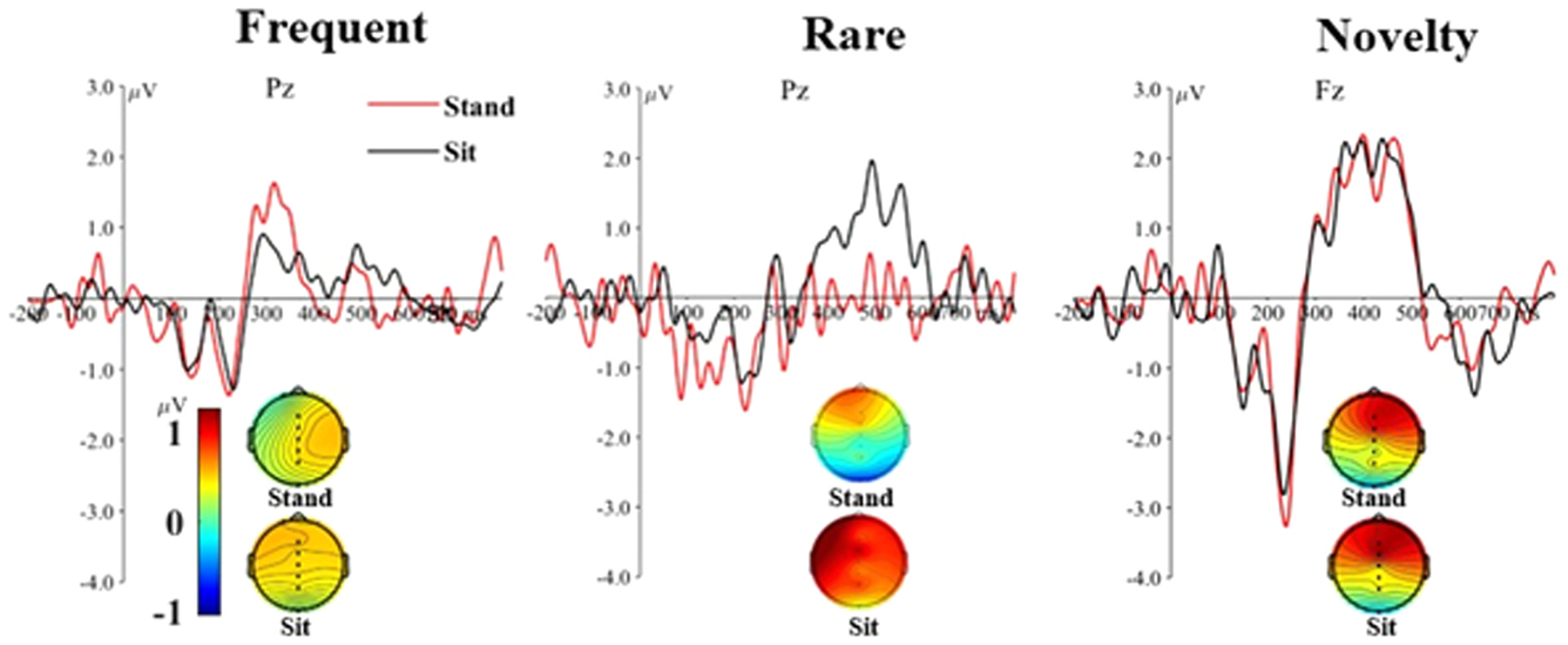
Grand average waveforms of frequent and rare responses at channel Pz, the novelty responses at channel Fz, and scalp maps of the mean P3 amplitude (250 – 550 ms), for the standing and sitting conditions.

Fig. 2 shows the grand averages of the frequent and rare P3b responses at channel Pz, the novelty P3a response at channel Fz, and the scalp maps of the P3 between 250 and 550 ms. No differences were observed in mean P3 amplitude of the frequent ERP between standing (0.43 ± 1.60 μV) and sitting (0.40 ± 1.11 μV) at channel Pz (p = 0.84). Mean P3b amplitude of the rare responses was significantly lower in the standing (− 0.06 ± 2.98 μV) compared to the sitting (0.65 ± 2.80 μV) condition at channel Pz (unadjusted p = 0.0007; adjusted p = 0.005 after controlling for age and sex). The difference wave (rare – frequent) also produced significant differences between standing (−0.50 ± 3.65 μV) and sitting (0.21 ± 3.10 μV) at channel Pz (unadjusted p = 0.02; adjusted p = 0.005). No differences were found in P3a amplitude between standing (1.00 ± 2.86 μV) and sitting (1.09 ± 2.31 μV) for the novelty trials at channel Fz (p = 0.42).

The P3 fractional area latency did not produce significant results in the standing compared to sitting position for the frequent responses (388.54 ± 36.53 ms vs 390.03 ± 35.06 ms; p = 0.75), rare responses (397.86.24 ± 36.91 ms vs 400.49 ± 36.71 ms; p = 0.39), and rare – frequent responses (398.70 ± 33.96 ms vs 400.30 ± 34.35 ms; p = 0.69) at channel Pz. Similarly, no differences were found in novelty responses between standing (390.03 ± 35.06 ms) and sitting (394.85 ± 34.69 ms) conditions at channel Fz (p = 0.41).

### 3.3. Relationship between P3b amplitude and reaction time across postural conditions

We conducted a linear mixed model to examine the relationship between the mean P3b amplitude at Pz and reaction time to target stimuli across conditions. For the rare stimulus, the results revealed a significant interaction between reaction time and condition (standing vs. sitting) in predicting mean P3b amplitude (β = −0.002, p = 0.03) as well as the difference wave (rare – frequent) (β = −0.002, p = 0.04) at channel Pz. However, for the frequent stimulus, no significant interaction effect was observed between reaction time and condition (standing vs. sitting) in predicting mean P3b amplitude (β = −0.002, p = 0.08) or the difference wave (rare – frequent) (β = −0.002, p = 0.20) at channel Pz.

### 3.4. Multivariate pattern analysis

**Fig. 3.**
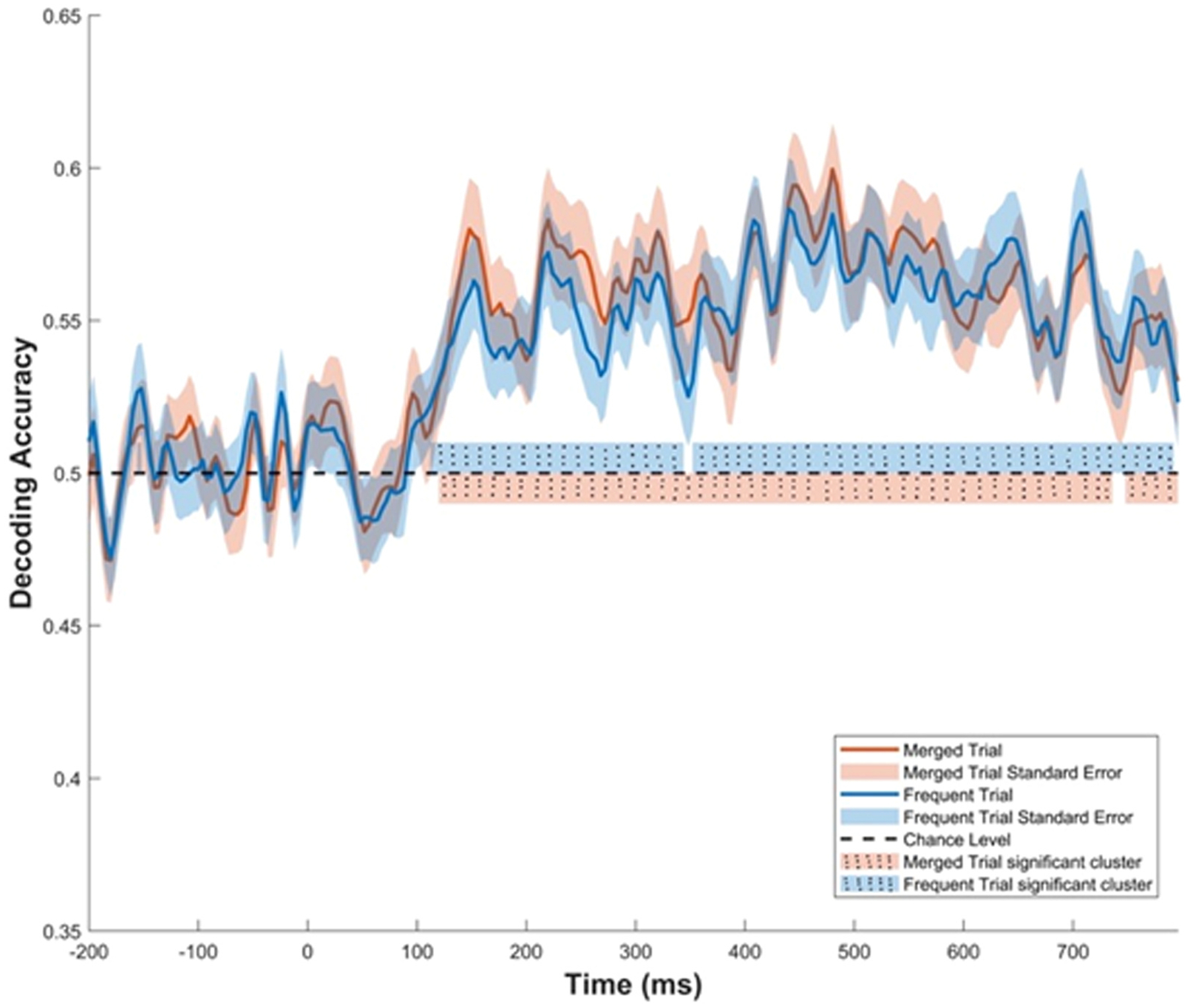
Multivariate pattern analysis results decoding the standing from sitting conditions. Blue shows the classification accuracy in the event-related potential of only frequent trials; Pink shows the classification accuracy in the event-related potential of the merged frequent, rare, and novelty trials.

The MVPA results are displayed in Fig. 3. The classification accuracy was better than chance in both the frequent only and the merged trials, with significance reached in a cluster ranging from around 100 ms until 800 ms post-stimulus. Specifically, the classification accuracy reached significance for the frequent trials between 112 ms and 344 ms post-stimulus and again between 352 ms and 792 ms post-stimulus. The MVPA on the merged trials identified significant time windows between 120 ms and 736 ms post-stimulus and again between 748 ms and 796 ms post-stimulus.

The confusion matrix between 250 and 550 ms (P3 component) showed that the model performed better predicting the sitting compared to the standing conditions (Table 3). The confusion matrix for the frequent and merged trials in the P3 window predicted the sitting condition with 59 ± 13 % accuracy, and the standing condition with 54 ± 15 % accuracy across both frequent and merged trials (p = 0.06). Similarly, in the significant cluster window between 100 ms and 800 ms post-stimulus, the model again showed better accuracy for predicting the sitting condition, with a 59 ± 12 % accuracy for merged trials and 58 ± 12 % for frequent trials (p = 0.04).

### 3.5. Data quality of ERP

Data quality for the mean amplitude was assessed using the aSME. At the Pz channel for frequent stimuli, the aSME was similar in standing and sitting conditions (0.82 ± 0.55 μV vs. 0.71 ± 0.51 μV; p = 0.18). The aSME values were also comparable for rare stimuli between standing and sitting conditions (2.02 ± 1.65 μV vs. 1.80 ± 1.44 μV; p = 0.28) at the Pz channel. Similarly, there were no differences in aSME values for novel stimuli between standing and sitting conditions at the Fz channel (1.74 ± 1.51 μV vs. 1.70 ± 1.14 μV; p = 0.85).

For fractional area latency measurements, data quality was evaluated using the bSME. At channel Pz for frequent stimuli, the bSME values were similar between standing and sitting conditions (26.07 ± 7.38 ms vs. 25.07 ± 7.63 ms; p = 0.33). Similarly, for rare stimuli at Pz, the bSME showed nearly identical data quality between standing and sitting (24.64 ± 11.79 vs. 24.67 ± 8.04 ms; p = 0.96). At the Fz channel, the bSME for novel stimuli was significantly lower in the standing condition compared to the sitting condition (21.33 ± 9.85 ms vs. 25.48 ± 9.69 ms; p = 0.002).

## 4. Discussion

This study provides neural evidence that maintaining postural control during standing reallocates attentional resources away from a concurrent cognitive task. The lower amplitude of the P3b subcomponent reflects the specific cognitive processes most impacted by the postural task. MVPA reveals that early sensory and late motor-related neural activities are also influenced by the demands of maintaining postural control.

### 4.1. Postural control reallocates attentional resources away from the cognitive dual-task

We measured attentional resource allocation using the P3 ERP in response to an auditory oddball task. This task included frequent, rare, and novelty stimuli, which produced a peak P3b at Pz for the rare stimuli at around 500 ms post-stimulus, and a peak P3a at Fz for the novelty stimuli at around 380 ms post-stimulus. Univariate analysis revealed a lower P3b amplitude for the rare stimuli and the rare – frequent stimuli in quiet standing compared to sitting. A lower P3 amplitude is thought to reflect increased cognitive workload [15], suggesting that the postural control needed for quiet standing takes away attentional resources from the cognitive task. We found a stronger, negative association between reaction time and P3b amplitude in standing compared to sitting. This results suggests that the relationship between reaction time and P3b amplitude varies depending on the postural condition, indicating that the postural demands of standing may reallocate attentional resources away from the cognitive task. In our experiment, the P3b response required a button press as soon as the frequent or rare stimulus was identified, creating a close temporal link between cognitive decision-making and motor execution (including motor planning, anticipatory movements, and pressing the button). Therefore, the P3 window of 250–550 ms may have temporarily overlapped with motor-related potentials. The average reaction times for the frequent stimuli (409 ± 158 ms for standing and 448 ± 159 ms for sitting) suggest that the P3 window may indeed have captured some motor-related activity. By subtracting the frequent from the rare ERP that both required a button press, we mitigated the potential influence of motor-related neural activity confounding the P3 ERP. The significant difference in rare – frequent ERP between standing and sitting result confirms that postural control affects the P3b.

### 4.2. Postural control affects conscious cognitive processing

While standing lowered P3b amplitude compared to sitting, the P3a was largely unaffected by the dual-task. There are key differences in the cognitive processes between the P3b and P3a that may explain this finding. The P3b component is closely linked to elaborate, conscious cognitive processes involved in stimulus evaluation, categorization, and context updating [15,45]. In contrast, the P3a reflects the engagement of attentional resources towards a new or unexpected event, signaling that the brain has detected a deviation from a predicted pattern. This rapid orientation to novelty represents the immediate, non-conscious response to unexpected stimuli, and does not require the extensive cognitive evaluation or decision-making of the P3b [15,45]. Our results demonstrate that postural control particularly influences higher-order cognitive processes that require conscious processing, rather than automated cognitive processes that demand less cortical control.

While previous studies have precisely located the dorsolateral prefrontal cortex (DLPFC) as a cortical area that activates when processing dual-tasks [46], our ERP study pinpoints the exact cognitive processes that are immediately affected by a dual cognitive-postural control task. The link between DLPFC activity and P3 has also been established in transcranial direct stimulation (tDCS) studies [47]. Anodal tDCS application over the left DLPFC increased parietal P3b amplitude by enhancing the functional connectivity in the theta and delta bands between the frontal and parietal areas after a target stimulus requiring categorization and context updating [47]. Our study and tDCS studies therefore confirm the theoretic P3 generation model that attention and memory processes are controlled by interregional communication of relevant information in the frontoparietal network during oddball paradigms [15].

### 4.3. Postural control affects neural activity beyond the P3 window

MVPA yielded classification accuracies surpassing chance levels for both frequent and merged trials. The decoding was successful in predicting 59 % of the sitting ERP’s and 54 % of the standing ERP’s within the P3 time window. While these accuracy rates are modest, the aim of this study was not to develop a highly predictive model based on ERP’s. The decoding analysis reveals that there is information contained in the ERP signal that is influenced by the postural control of balance. In addition, the MVPA showed significant decoding accuracy beyond the P3 time window, demonstrating the power of machine learning approaches to reveal cortical dynamics that are not captured by univariate analyses. ERP signals earlier (100–250 ms) and later (550–800 ms) than the P3 window also successfully decoded the standing from the sitting trials, suggesting that there are distinguishable patterns in neural activity beyond the P3 window. The temporal sequence of the ERP to an auditory oddball task starts with the P1, a small positive deflection occurring around 50–100 ms after stimulus onset that reflects early sensory processing of the sounds. This component does not appear to be affected by the postural control task. The MVPA starts to successfully decode at 100 ms. The N1 component peaks at around 100 ms and signals early sensory and perceptual processes from the synchronization between the primary and secondary auditory cortices [48]. The P2 emerges at 150 ms and reflects perceptual processing related to the salience and difficulty of the oddball [49]. At around 200–250 ms, the N2 appears and is associated with conflict detection, stimulus categorization, and response inhibition [48]. After the P3 component, the ERP continues to show slow waves (SW), reflecting memory consolidation and motor response preparation [50]. The period after the motor response may indicate ongoing cognitive control, evaluation of the response, and preparation of the next trial. The effect of postural control on these ERP components should be investigated in future studies.

There are some limitations that could be addressed in future studies. First, the standing condition may have increased the signal-to-noise ratio. The percentage of rejected epochs in the standing condition (14.59 %) compared to the sitting condition (3.41 %) suggests that standing did introduce additional noise to the EEG signals. However, after removing these artifacts, the data quality of the P3 epochs in standing was similar (and even better for the novelty trials) to the data quality in sitting conditions, and comparable to benchmark values reported in another study [51]. Visual inspection of the pre-stimulus grand average ERP waveforms also did not reveal more noise in the standing condition compared to the sitting conditions. Second, the study design included only 40 frequent, 5 rare, and 5 novelty trials per participant, which might be insufficient for measuring the P3b component with the same precision as would be possible with more trials, particularly for the rare and novelty stimuli. We adhered to the 80 % frequent:10 % rare:10 % novelty rule of oddball oddball task to maintain the standard balance across conditions [52]. This decision was also made to minimize potential participant fatigue, given the standing tasks were limited to 2 min. To mitigate the effect of the low trial numbers, we offset this with a larger sample size, which enhances the reliability of the grand-averaged ERPs despite the few trials. Additionally, the signal quality measures (aSME and bSME) showed no significant differences between conditions and were consistent with benchmark values, supporting the adequacy of the data quality. Third, we used a low-density 8-channel EEG system. Although the system increased comfort and convenience, subtle variations in neural activity across cortical regions may have been missed in the MVPA due to the fewer channels. Also, although we excluded participants with neurological or musculoskeletal conditions affecting balance, we did not formally assess baseline postural stability, which may contribute to variability in attentional demands during standing. Lastly, the age of our participants ranged from 18 to 88 years, covering a wide spectrum from young adults to older adults. Given that age influences P3 ERP amplitude in auditory oddball tasks [52], there may have been age-related effects in the univariate and multivariate analyses. We plan to explore the effects of aging on the P3 ERP during different postural control conditions in future studies.

### 4.4. Conclusion

In conclusion, this study provides neural evidence that maintaining quiet standing demands attentional resources, particularly impacting the conscious cognitive processes involved in decision-making, as reflected by the lower P3b amplitude during standing compared to sitting. MVPA further revealed distinct neural patterns related to postural control across different ERP components, emphasizing the need for future research to explore these effects in more detail and across different age groups.

## Figures and Tables

**Table 1:** Description of demographic variables (N = 126).

Variable	Description
Age, years	61.34 ± 19.61
Sex, female:male	86 (68 %); 40 (32 %)
Race, White:Black:Asian:Hispanic:Mixed	108 (85.7 %): 9 (7.1 %): 6 (4.8 %): 2 (1.6 %):1 (0.8 %)
Education, years	17.30 ± 3.01
Montreal Cognitive Assessment, /30	28.86 ± 1.01
Variables are described as mean ± standard deviation or frequency (percentage).	

**Table 2:** Behavioral performance on the auditory oddball task.

Measure	Standing	Sitting	t value	P value
Frequent stimuli, correct responses (/40)	36 ± 8	33 ± 12	1.83	0.07
Frequent stimuli, reaction time (ms)	409 ± 158	448 ± 159	−1.58	0.11
Rare stimuli, correct responses (/5)	4 ± 1	4 ± 1	0.59	0.55
Rare stimuli, reaction time (ms)	595 ± 195	540 ± 157	1.92	0.06

**Table 3:** Confusion matrix for the average decoding accuracy between 250 and 550 ms (P3 ERP) and between 100 ms and 800 ms post-stimulus (significant cluster window).

		P3 window (250 – 550 ms)				Significant cluster window (100 ms – 800 ms)			
		Frequent trials True labels		Merged trials True labels		Frequent trials True labels		Merged trials True labels	
		Standing	Sitting	Standing	Sitting	Standing	Sitting	Standing	Sitting
**Predicted labels**	**Standing**	0.54 ± 0.15	0.41 ± 0.13	0.54 ± 0.15	0.41 ± 0.13	0.53 ± 0.13	0.42 ± 0.12	0.53 ± 0.14	0.41 ± 0.12
	**Sitting**	0.46 ± 0.15	0.59 ± 0.13	0.46 ± 0.15	0.59 ± 0.13	0.47 ± 0.003	0.58 ± 0.12	0.47 ± 0.14	0.59 ± 0.12

## Data Availability

Data will be made available on request.
